# Gene mutation profiling in microsatellite instability colorectal cancer and its association with the efficacy of immunotherapy: A retrospective study

**DOI:** 10.1002/cam4.6910

**Published:** 2024-05-15

**Authors:** Ying Liu, Kang Cui, Wang Ma

**Affiliations:** ^1^ Department of Oncology The First Affiliated Hospital of Zhengzhou University Zhengzhou Henan People's Republic of China

**Keywords:** colorectal cancer, gene profiling, immunotherapy, microsatellite instability‐high, predictive marker

## Abstract

**Background:**

Microsatellite instability‐high (MSI‐H) colorectal cancer (CRC) is known for its heightened responsiveness to immunotherapy. However, establishing robust predictive markers for immunotherapy efficacy remains imperative. This retrospective study aimed to elucidate the genetic landscape of MSI‐H CRC and correlate these genetic alterations with immunotherapy outcomes in a cohort of 121 patients.

**Methods:**

We analyzed clinical and molecular data from 121 patients with MSI‐H CRC. We conducted a thorough genetic analysis of MSI‐H CRC patients, with a specific emphasis on the APC, TP53, RAS, and MMR genes. We further analyzed the relationship between gene mutations and immunotherapy efficacy. The primary endpoints analyzed were objective response rate (ORR) and progression‐free survival (PFS). All statistical analysis was conducted using SPSS26.0 and R 4.2.0 software.

**Results:**

Our findings underscored the complexity of the genetic landscape in MSI‐H CRC, shedding light on the intricate interplay of these genes in CRC development. Notably, mutations in MMR genes exhibited a distinctive pattern, providing insights into the underlying mechanisms of MSI‐H. Furthermore, our results revealed correlations between specific genetic alterations and immunotherapy outcomes, with a particular focus on treatment response rates and progression‐free survival.

**Conclusion:**

This study represents a significant step toward unraveling the genetic nuances of MSI‐H CRC. The distinctive pattern of MMR gene mutations not only adds depth to our understanding of MSI‐H CRC but also hints at potential avenues for targeted therapies. This research sets the stage for future investigations aimed at refining therapeutic strategies and improving outcomes for patients with MSI‐H CRC.

## 1 INTRODUCTION

Colorectal cancer is the most frequently occurring malignant tumor in the digestive system, and its global incidence is on the rise.^1^ It is the second leading cause of cancer‐related deaths in developed countries, with the number of newly diagnosed cases reaching approximately 2 million. With the ongoing advancements in scientific investigation, notable improvements have been observed in the prognosis of colorectal cancer. In the realm of colorectal cancer treatment, current treatment options for colorectal cancer encompass surgery, radiotherapy, chemotherapy, and targeted therapy.^2^ Among these, targeted therapy has made significant progress in the treatment of relapsed or refractory cases.^3^ Additionally, a distinct molecular subtype of colorectal cancer has been identified, characterized by high microsatellite instability (MSI‐H).^4^ Further understanding of the pathogenesis of colorectal cancer has revealed significant differences in pathological features, molecular characteristics, and clinical prognosis in colorectal cancer with high levels of tumor microsatellite instability (MSI‐H).^5^

MSI‐H colorectal cancer, accounting for approximately 15% of all cases, exhibits a high level of microsatellite instability.^6^, ^7^ Microsatellite instability (MSI) in colorectal cancer is characterized by genetic variations, characterized primarily by the instability of microsatellite sequences (short tandem repeats, STRs) within DNA, typically caused by defects in DNA mismatch repair (MMR) genes.^8^, ^9^ Studies have shown that MSI‐H colorectal cancer patients respond well to immunotherapy.^10^, ^11^, ^12^ Immunotherapy achieves its therapeutic efficacy by inhibiting the PD‐1/PD‐L1 signaling pathway or by activating T‐cell antitumor responses, making it a safer and sustainable treatment option.^13^ In recent clinical trials, anti‐PD‐1 monoclonal antibody and anti‐PD‐L1 monoclonal antibody have shown promising therapeutic effects in patients with MSI‐H colorectal cancer.^14^, ^15^

In addition, MSI‐H colorectal cancer may enhance the efficacy of immunotherapy through a series of molecular alterations. For example, it exhibits a higher tumor neoantigen burden, which enables the immune system easier to detect and recognize these tumor cells.^7^, ^16^ Additionally, MSI‐H colorectal cancer is characterized by increased tumor‐infiltrating lymphocyte, which may further enhance the efficacy of immunotherapy.^13^ Clinical data have demonstrated an overall response rate of approximately 50% to 60% to immune checkpoint inhibitors in patients with MSI‐H colorectal cancer, highlighting the heterogeneity between patients or tumors.^14^, ^15^, ^17^ By exploring the heterogeneous factors in tumors or patients that are associated with the efficacy of immunotherapy, it provides valuable guidance for precise and personalized clinical treatment, thereby achieving the optimal impact of immunotherapy. Therefore, it is important to study the relationship between the heterogeneity of MSI‐H colorectal cancer and immunotherapy for the development of personalized treatment approaches.

The aim of this study was to analyze the predictive effect of clinical characteristics in MSI‐H colorectal cancer patients for their response to immunotherapy, aiming to provide new insights and a basis for individualized treatment of MSI‐H colorectal cancer. We retrospectively analyzed the clinical data of a group of patients with MSI‐H colorectal cancer to study their clinical characteristics and the impact of immunotherapy on their prognosis. By collecting the basic information of the patients, treatment plans, curative effect, and prognosis, we aimed to contribute to the development of individualized treatment of MSI‐H colorectal cancer. However, it should be noted that some data were missing for various reasons. Nevertheless, we made every effort to utilize the available data for analysis and interpretation.

## 2 METHODS

### 2.1 Patients

This study included a cohort of 121 patients with MSI‐H colorectal cancer at the first affiliated hospital of Zhengzhou University from January 2019 to November 2023, as confirmed by NGS testing. Inclusion criteria: (1) Patients aged 18 years or older with colorectal cancer; (2) histopathology confirmation of MSI‐H colorectal cancer; (3) ability to comply with the study protocol; (4) adequate medical history and clinical evaluation; and (5) ability to provide relevant clinical, pathological, and molecular data. Exclusion criteria were as follows: (1) Patients below the age of 18; (2) presence of other serious complications or diseases, such as severe heart disease, liver and kidney damage, which could potentially affect the safety or efficacy of immunotherapy; (3) pregnant or lactating women; and (4) patients with severe immune system defects or immunosuppressive status, such as HIV‐infected people or organ transplant patients (Figure S1). Data on clinicopathological features, tumor genomics, and outcomes were collected. We assessed response to PD‐1/L1‐targeted therapy according to the use of RECIST 1.1 guidelines. This study was approved by Ethics Committee of The First Affiliated Hospital of Zhengzhou University (approval number: 2023‐KY‐0604‐002).

### 2.2 Statistical analysis

Collected data were analyzed using statistical package IBM SPSS Statistics 26.0 software (SV26|IBM SPSS Statistics 26) and R 4.2.0. Continuous variables were expressed as median (range), while categorical variables as absolute numbers. Chi‐square test and the Student's t‐test were employed for comparing categorical and continuous variables, respectively. Kaplan–Meier method was used to estimate cumulative survival curves and median progression‐free survival (PFS) time. Differences between survival curves were assessed using the two‐tailed log‐rank test for statistical significance. Multivariate prognostic analysis of PFS was estimated using Cox proportional hazards model. A significance level of 0.05 was used to determine statistical significance.

## 3 RESULTS

### 3.1 Patient characteristics

We collected detailed clinical information from 121 patients diagnosed with colorectal cancer (CRC) exhibiting microsatellite instability‐high (MSI‐H). Clinical and tumor genomic characteristics of the cohort are summarized in Table 1. Of these patients, 54 were treated with immune checkpoint inhibitors, including 32 responders (CR/PR) and 22 non‐responders (SD/PD). Median follow‐up time was 29 months (95% CI: 22.74–35.25). The median age of the patients was 51 years (range 19–79 years), with 61.2% of them being male.

**TABLE 1:** The clinicopathological characteristics and gene mutation profiling of patients.

Characteristics	*n* (%) or median (range)	Characteristics	*n* (%) or median(range)
Age (year)		MSH6 mutation	
Median	51	Yes	31 (46.3%)
Range	(19–79)	No	36 (53.7%)
Gender		MSH2 mutation	
Male	74 (61.2%)	Yes	14 (20.9%)
Female	47 (38.8%)	No	53 (79.1%)
TMB		MLH1 mutation	
Median	80.90	Yes	17 (25.4%)
Range	(15.25–386.20)	No	50 (74.6%)
Stage		PMS2 mutation	
I+II+III	92 (76.0%)	Yes	12 (17.9%)
IV	29 (24.0%)	No	55 (82.1%)
KRAS mutation		BRCA2 mutation	
Yes	57 (53.8%)	Yes	14 (21.9%)
No	49 (46.2%)	No	50 (78.1%)
NARS mutation		TP53 mutation	
Yes	2 (1.9%)	Yes	9 (14.1%)
No	104 (98.1%)	No	55 (85.9%)
BRAF mutation		APC mutation	
Yes	6 (5.7%)	Yes	25 (39.1%)
No	100 (94.3%)	No	39 (60.9%)
Pik3ca mutation		Arid1a mutation	
Yes	22 (21.2%)	Yes	24 (44.4%)
No	82 (78.8%)	No	30 (55.6%)

### 3.2 Gene mutation profile in MSI‐H colorectal cancer

**FIGURE 1:**
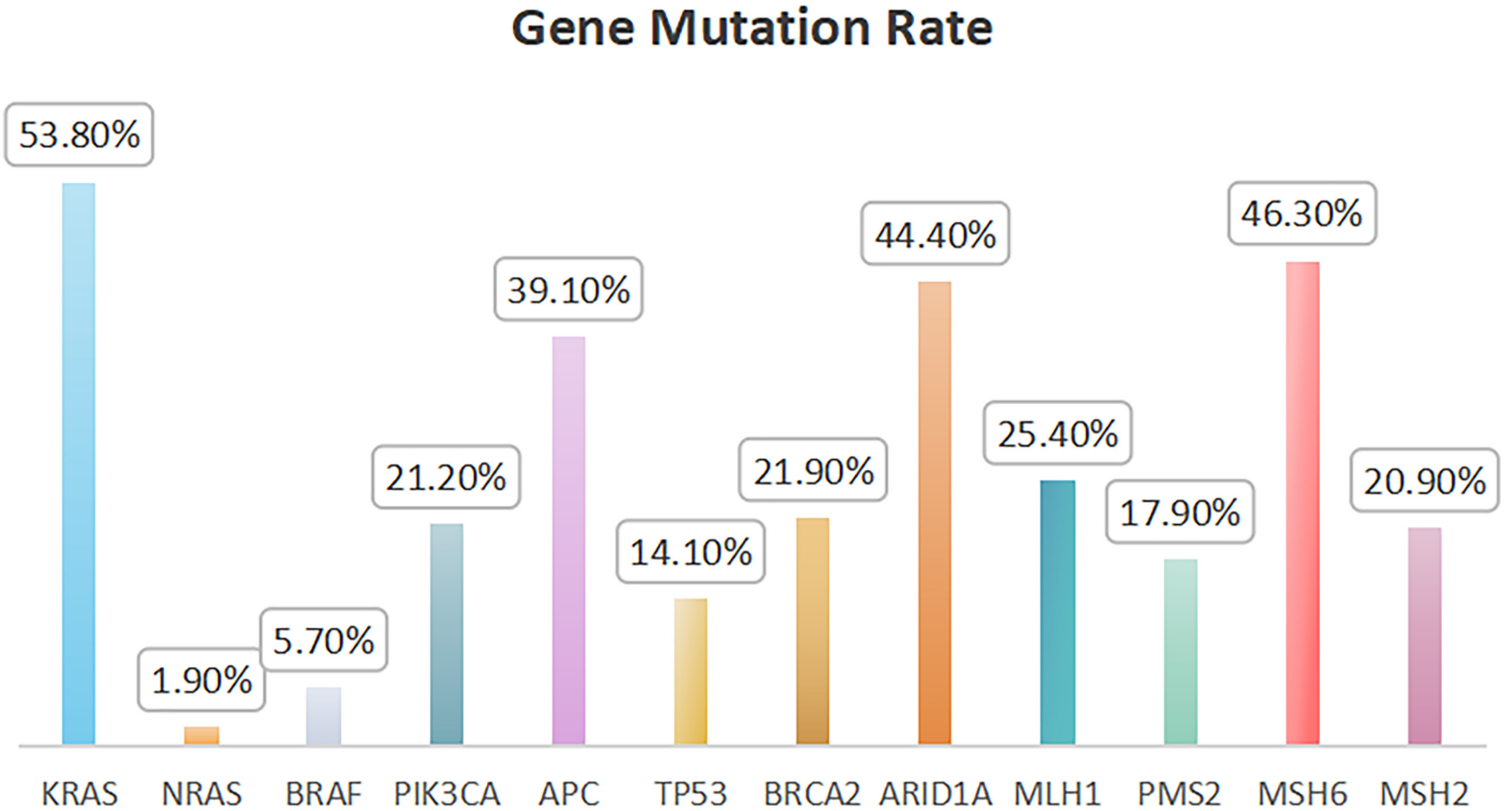
Correlation among different clinical feature variables.

We conducted a comprehensive analysis of the mutation status in 121 patients with MSI‐H colorectal cancer (Figure 1). The mutational landscape reveals KRAS mutations as the most prevalent, constituting 53.8% of the total. PIK3CA mutations were observed in 22 cases, accounting for 21.1%, while BRAF and NRAS mutations occurred in 6 cases (5.7%) and 2 cases (1.9%), respectively. Within the APC and TP53 genes, mutational frequencies were notable, with 25 cases (39.1%) exhibiting APC mutations and 9 cases (14.1%) showing TP53 mutations. BRCA2 mutations were present in 14 cases, representing 21.9%, and ARID1A mutations occurred in 24 cases, constituting 44.4%. Examining DNA mismatch repair genes, the mutation frequencies of MSH2, MSH6, PMS2, and MLH1 were significantly higher compared to their frequencies in MSS colorectal cancer. MSH6 mutations were the most prevalent, occurring in 31 cases (46.3%), followed by 17 cases (25.4%) with MLH1 mutations, 14 cases (20.9%) with MSH2 mutations, and 12 cases (17.9%) with PMS2 mutations (Table 1).

### 3.3 Correlation analysis among the 10 variables

**FIGURE 2:**
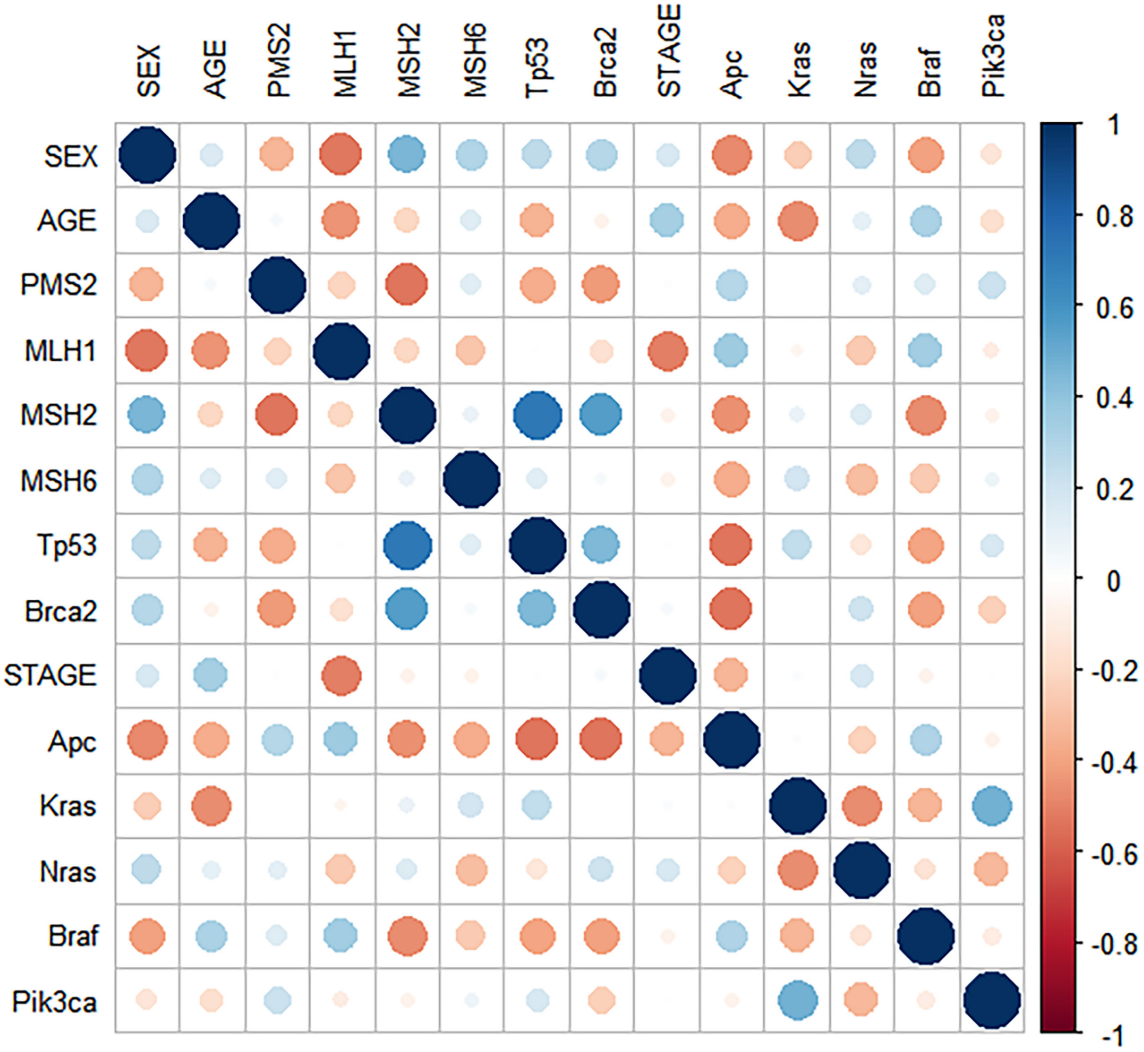
Gene mutation profiling in MSI‐H colorectal cancer.

To assess collinearity among the selected variables, we conducted correlation analysis using the corrplot package in R 4.1.3. Among the nine independent variables, no significant collinearity was observed (Figure 2). The chi‐square and *p*‐values showed no significant difference in the composition ratio of the variables among the subgroups (Table 2).

**TABLE 2:** Correlation between MSH6 mutation and clinical characteristics.

Factor	MSH6 mutation		χ^2^	*p*‐value
	MSH6 wild	MSH6 mut	0.033	0.856
Stage				
I+II+III	26	23		
IV	10	8		
Sex			1.692	0.193
Female	16	9		
Male	20	22		

### 3.4 Survival analysis

**FIGURE 3:**
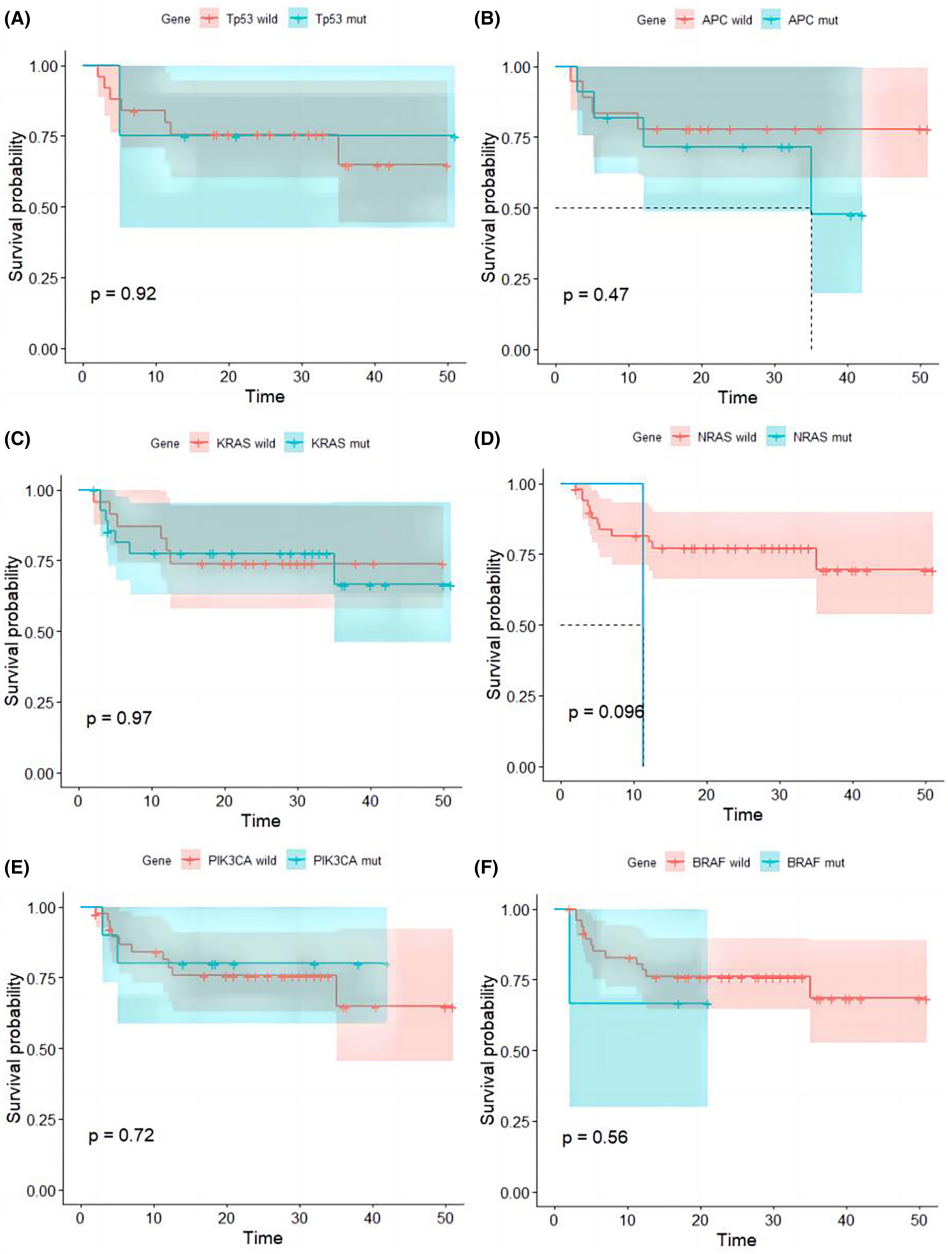
Kaplan–Meier survival curve of mutation patterns associated with PFS. (A) Kaplan–Meier survival curve of Tp53 mutation, (B) Kaplan–Meier survival curve of APC mutation, (C) Kaplan–Meier survival curve of KRAS mutation, (D) Kaplan–Meier survival curve of NRAS mutation, (E) Kaplan–Meier survival curve of Pik3CA mutation, (F) Kaplan–Meier survival curve of BRAF mutation.

**FIGURE 4:**
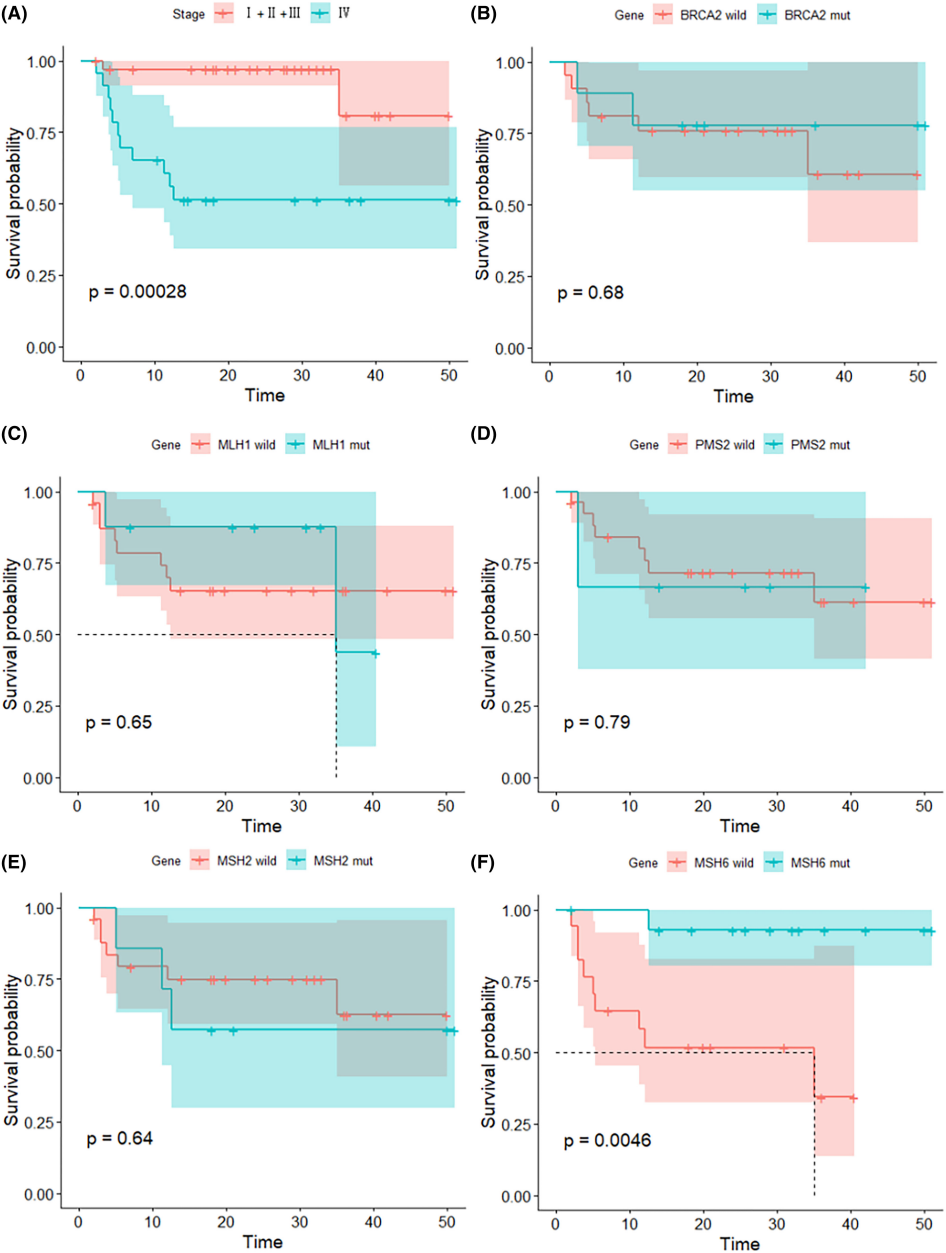
Kaplan–Meier survival curve of stage and mutation patterns associated with PFS. (A) Kaplan–Meier survival curve of Stage, (B) Kaplan–Meier survival curve of BRCA2 mutation, (C) Kaplan–Meier survival curve of MLH1 mutation, (D) Kaplan–Meier survival curve of PMS2 mutation, (E) Kaplan–Meier survival curve of MSH2 mutation, (F) Kaplan–Meier survival curve of MSH6 mutation.

The primary objective of this study was to assess progression‐free survival (PFS). We performed survival analysis to examine the association between different clinical characteristics, molecular features of colorectal cancer patients, and their PFS using the log‐rank test. In terms of predicting PFS, among all tested variables associated with progression‐free survival (PFS) using Cox regression model, age, gender, lymph node metastasis (LNM), RAS status, and BRAF status did not affect PFS. Tumor stage and MSH6 mutation status were found to be significant predictors of PFS (*p* < 0.05) (Table 3). Subsequently, we included these two variables in a multivariable Cox regression analysis (Table 4), which also indicated their significance in predicting PFS (*p* < 0.05) (Figures 3 and 4).

**TABLE 3:** Univariate Cox regression analyses of variables associated with PFS.

Factor	HR (95% CI)	*p*‐value	Factor	HR (95% CI)	*p*‐value
Age (year)	1.01 (0.97–1.04)	0.763	MSH6 mutation Yes versus No	0.09 (0.01–0.73)	0.024
Gender Male versus Female	0.74 (0.24–2.26)	0.592	MSH2 mutation Yes versus No	1.38 (0.36–5.34)	0.641
TMB	0.99 (0.97–1.01)	0.537	MLH1 mutation Yes versus No	0.70 (0.15–3.30)	0.652
Stage IV versus I+II+III	9.75 (2.16–44.14)	0.003	PMS2 mutation Yes versus No	1.24 (0.26–5.86)	0.786
KRAS mutation Yes versus No	1.02 (0.34–3.06)	0.968	BRCA2 mutation Yes versus No	0.71 (0.14–3.54)	0.678
NARS mutation Yes versus No	4.87 (0.62–38.45)	0.133	TP53 mutation Yes versus No	0.90 (0.11–7.33)	0.922
BRAF mutation Yes versus No	1.81 (0.23–14.07)	0.569	APC mutation Yes versus No	1.66 (0.41–6.62)	0.476
Pik3ca mutation Yes versus No	0.76 (0.17–3.47)	0.722	Arid1a mutation Yes versus No	0.58 (0.10–3.45)	0.545

**TABLE 4:** Multivariate Cox regression analyses of variables associated with PFS.

Factor	HR	95% CI	*p*‐value
Stage before treatment IV versus I/II/III	11.81	1.985–70.264	0.007
MSH6 mutation Yes versus No	0.04	0.004–0.413	0.006

### 3.5 Connecting MSH6 mutation with objective response

We initially analyzed the association between the clinical and molecular characteristics of the patients and their objective response (OR) to immunotherapy. The findings revealed significant differences in the distribution of responders (CR/PR) and non‐responders (SD/PD) among different subgroups of the pretreatment stage and Msh6 mutation status. However, the distribution of objective response rate tended to be the same across different subgroups of other variables, indicating that MSH6 mutation and pretreatment stage may serve as biomarkers for predicting immunotherapy response (Table 5).

**TABLE 5:** Correlation between variables and treatment response.

Factor	Treatment response		χ^2^	*p*‐value
	CR/PR	SD/PD	0.064	0.801
Stage				
I+II+III	20	13		
IV	12	9		
MSH6 mutation			16.941	﹤0.001
Yes	15	0		
No	5	12		

### 3.6 Visualization of Cox regression model

**FIGURE 5:**
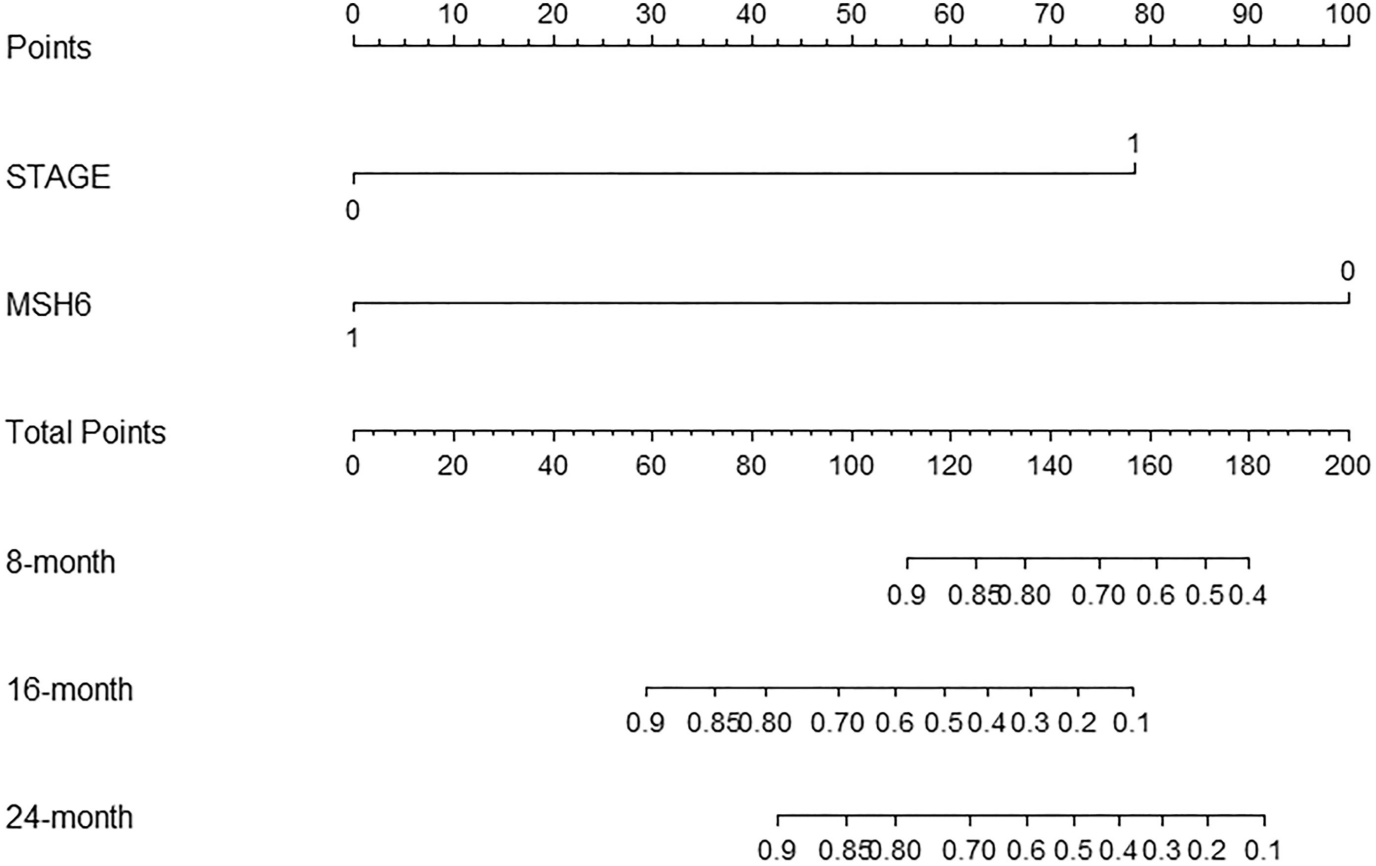
Nomogram is build to predict the progression‐free survival.

To visualize the Cox regression model, we employed R language to create a nomogram that can predict the efficacy of immunotherapy in MSI‐H colorectal cancer patients. This nomogram can be used to predict the response of immunotherapy more conveniently and intuitively, expanding its potential application in clinical practice (Figure 5).

## 4 DISCUSSION

Recent advancements in high‐throughput Sequencing technology, particularly next‐generation sequencing (NGS), have provided us with more comprehensive, accurate, and efficient analytical methods. The application of NGS can not only detect gene mutations in colorectal cancer tissues, but also evaluate Microsatellite Instability (MSI) and other important indicators. The widespread application of NGS has bridged the gap between pathological tissue alterations and micro‐level molecular changes in tumors. Our understanding of the pathogenesis and treatment mechanisms of colorectal cancer is now advancing toward the molecular level.^18^, ^19^, ^20^ Personalized therapy is a strategy that involves developing targeted treatment plans based on the molecular characteristics and genetic variations of individual patients. By exploring the molecular mechanism, we can deeply understand the pathobiological characteristics of colorectal cancer, which in turn provides a scientific basis for individualized treatment strategies.^3^, ^21^

The statistical analysis reveals a potentially distinct mutational landscape in MSI‐H colorectal cancer compared to MSS colorectal cancer. While genes like KRAS, APC, and PIK3CA maintain mutation frequencies relatively consistent with MSS colorectal cancer, the most significant observation is the markedly higher mutation frequency of DNA mismatch repair (MMR)‐related genes in MSI‐H colorectal cancer compared to MSS colorectal cancer. Mismatch repair (MMR) genes, crucial for correcting DNA base mismatches, play a role in maintaining genome stability and reducing the likelihood of spontaneous mutations. However, in the context of deficient MMR (dMMR), where mismatch repair proteins are lacking, the phenomenon of high microsatellite instability remains unrepaired, leading to a highly microsatellite‐instable state. It is also noteworthy that the mutation frequency of ARID1A gene was also significantly higher in MSI‐H colon cancer than in MSS colon cancer (6.7%).^22^ The study indicates that ARID1A interacts with the mismatch repair (MMR) protein MSH2. ARID1A recruits MSH2 to chromatin during DNA replication, facilitating MMR. Therefore, ARID1A mutations may impair MMR and increase the mutation rate.^23^ This research suggests a unique genetic signature in MSI‐H colorectal cancer, emphasizing the importance of MMR‐related genes and revealing a potential role for ARID1A in influencing MMR processes. These findings contribute to a better understanding of the distinct molecular characteristics of MSI‐H colorectal cancer, with implications for targeted therapeutic strategies.

The MMR genes, including MSH6, MSH2, PMS2, and MLH1, play a crucial role in the mismatch repair system. We aimed to explore the relationship between MMR genes and the efficacy of immune therapy in MSI‐H colorectal cancer, and thus conducted a statistical analysis. Our analysis revealed a correlation between MSH6 gene status and both progression‐free survival (PFS) and objective response rate (ORR) in patients with MSI‐H colorectal cancer who underwent immune therapy, suggesting that MSI‐H colorectal cancer patients with MSH6 mutations exhibited better response to immune therapy. However, no significant differences in PFS and ORR were observed in relation to the mutation status of other MMR genes, including MSH2, PMS2, and MLH1. MSH6 is a critical protein in the DNA repair process, forming a stable hMutSα mismatch binding complex by interacting with MSH2. After recognizing mismatched base pairs, the MutSα complex recruits PMS2 and MLH1 proteins, forming the MutSβ complex, which corrects mismatched bases and insertion/deletion structures during DNA replication.^24^, ^25^ MSH6 possesses a highly disordered N‐terminal domain distinct from MSH2, and research has identified several functions that specifically require the N‐terminal domain of MSH6. The N‐terminal domain interacts with PCNA, which regulates the direction of MutSαcomplex to initiate mismatch repair.^26^ Additionally, the N‐terminal domain contains a nuclear localization sequence and participates in the transport of the MutSα complex.^27^ Phosphorylation sites on the N‐terminal domain also regulate MMR activity.^28^ The MutSα complex formed by MSH6 and MSH2 is also involved in other DNA repair processes, including base excision repair, transcription‐coupled repair, and double‐strand break repair.^29^ These findings highlight the importance of MSH6 in DNA repair processes. The favorable response of MSI‐H colorectal cancer patients with high microsatellite instability to immune therapy may be related to the high tumor antigen load and increased infiltration of lymphocytes. However, the specific signaling mechanisms that influence these characteristics in MSI‐H colorectal cancer remain unclear. Therefore, the response mechanism of the MSH6 gene to immune therapy in MSI‐H colorectal cancer patients requires further exploration. Based on our analysis results, we will conduct additional basic experimental research to elucidate the molecular‐level mechanisms involved.

With respect to limitations, it should be noted that this study was conducted with a retrospective study design, and there may be risks of a longer time span for data collection, patient bias. In addition, the relatively small sample size and pathological tissue from a single laboratory may limit the generalizability and external validity of our results. However, our study is the first to show the predictive value of MSH6 mutation status as a decision‐making tool in immune checkpoint inhibitor (ICPI) therapy for individuals with MSI‐H. These data provide a potential explanation for the differences in immune checkpoint inhibitor responses observed in MSI‐H colorectal cancer clinical trials and support the use of MSH6 mutation status for determining the sequence of checkpoint inhibitors and chemotherapy. The innovation of our study lies in linking the molecular characteristics of MSI‐H colorectal cancer immunotherapy and providing evidence for individualized treatment. By focusing on the molecular response of MSIH colorectal cancer to immunotherapy, our study provides a new perspective and theoretical basis for understanding the mechanisms underlying the response of colorectal cancer to immunotherapy. The integration of genetic mutations, Microsatellite Instability, and other clinical features provides new insights into the mechanisms of drug resistance and efficacy of immunotherapy at the molecular level.

## 5 CONCLUSION

According to our study, MSH6 gene is associated with tumor stage and immunotherapy response in patients with MSI‐H colorectal cancer. This finding suggests that MSH6 gene may influence the efficacy of immunotherapy for MSI‐H colorectal cancer. This provides a new research direction for us to explore the molecular mechanisms of immunotherapy and drug resistance in colorectal cancer patients.

## AUTHOR CONTRIBUTIONS

**Ying Liu:** Data curation (equal); formal analysis (equal); resources (equal); software (equal); writing – original draft (equal). **Kang Cui:** Conceptualization (equal); writing – review and editing (equal). **Wang Ma:** Supervision (equal); writing – review and editing (equal).

## FUNDING INFORMATION

This research was sponsored by National Natural Science Foundation of China (82073168).

## CONFLICT OF INTEREST STATEMENT

The author reports no conflicts of interest in this work.

## INSTITUTIONAL REVIEW BOARD STATEMENT

The study was reviewed and approved by the Ethics Committee of The First Affiliated Hospital of Zhengzhou University (2023‐KY‐0604‐002).

## INFORMED CONSENT STATEMENT

All study participants, or their legal guardians, provided written informed consent for personal and medical data collection prior to study enrolment.

## Supporting information


Figure S1.

## Data Availability

The data that support the findings of this study are available from the corresponding author upon reasonable request. Due to privacy and ethical considerations, some restrictions may apply to the availability of certain sensitive or confidential data. Raw data, as well as any additional information required to reproduce the results or conclusions of this study, will be made available to qualified researchers upon request.
