# Supplementary figures and images for: Gene mutation profiling in microsatellite instability colorectal cancer and its association with the efficacy of immunotherapy: A retrospective study
Source: Cancer Med. 2024 May 15;13(9):e6910. doi: 10.1002/cam4.6910 (PMC11094515; doi:10.1002/cam4.6910)

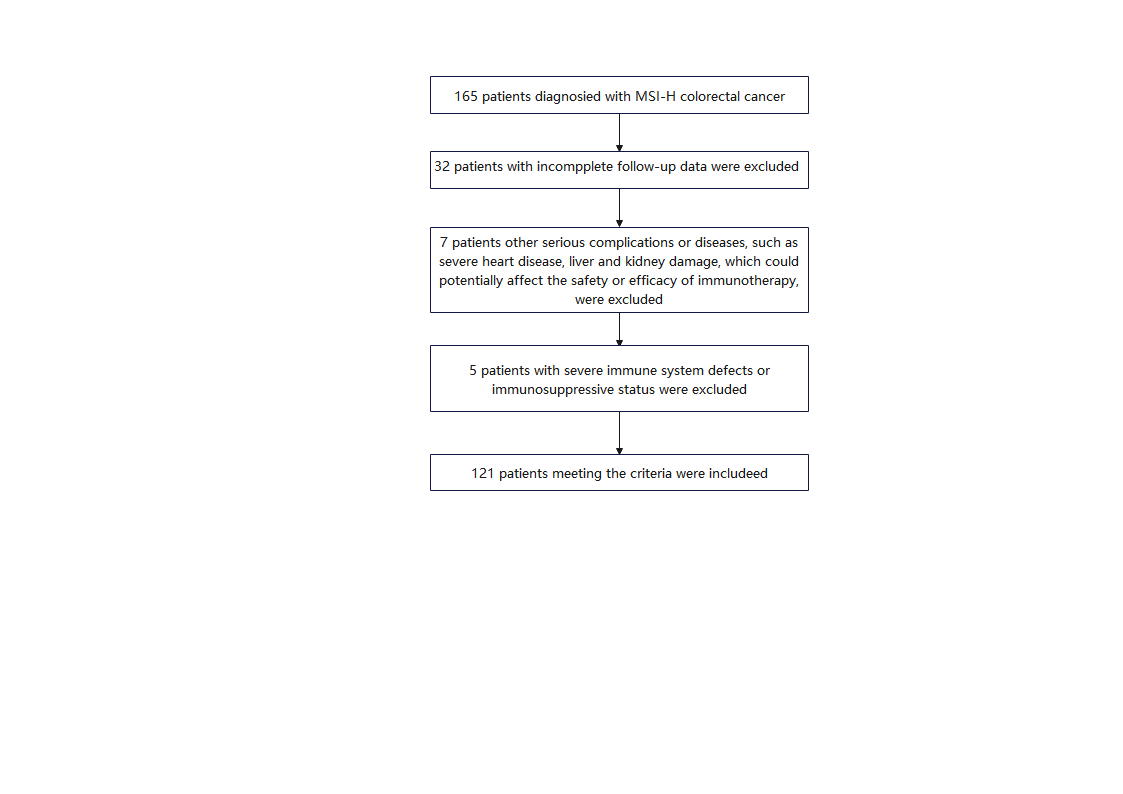

Supplement: Supplementary file 1 — Figure S1. [file CAM4-13-e6910-s001.tiff]
